# The sweet spot between predictability and surprise: musical groove in brain, body, and social interactions

**DOI:** 10.3389/fpsyg.2022.906190

**Published:** 2022-08-09

**Authors:** Jan Stupacher, Tomas Edward Matthews, Victor Pando-Naude, Olivia Foster Vander Elst, Peter Vuust

**Affiliations:** ^1^Center for Music in the Brain, Department of Clinical Medicine, Aarhus University and The Royal Academy of Music Aarhus/Aalborg, Aarhus, Denmark; ^2^Institute of Psychology, University of Graz, Graz, Austria

**Keywords:** music, entrainment, rhythm, movement, dance, Parkinson’s, predictive coding, syncopation

## Abstract

Groove—defined as the pleasurable urge to move to a rhythm—depends on a fine-tuned interplay between predictability arising from repetitive rhythmic patterns, and surprise arising from rhythmic deviations, for example in the form of syncopation. The perfect balance between predictability and surprise is commonly found in rhythmic patterns with a moderate level of rhythmic complexity and represents the sweet spot of the groove experience. In contrast, rhythms with low or high complexity are usually associated with a weaker experience of groove because they are too boring to be engaging or too complex to be interpreted, respectively. Consequently, the relationship between rhythmic complexity and groove experience can be described by an inverted U-shaped function. We interpret this inverted U shape in light of the theory of predictive processing and provide perspectives on how rhythmic complexity and groove can help us to understand the underlying neural mechanisms linking temporal predictions, movement, and reward. A better understanding of these mechanisms can guide future approaches to improve treatments for patients with motor impairments, such as Parkinson’s disease, and to investigate prosocial aspects of interpersonal interactions that feature music, such as dancing. Finally, we present some open questions and ideas for future research.

## Introduction

In the field of musicology, the term groove was coined in the context of African-American musical genres, such as R&B, jazz, soul, disco, funk, and hip-hop, where it can refer to esthetic qualities of the music, specific rhythmic patterns, or the musicians’ way of effortlessly synchronizing and interacting with each other (Senn et al., 2019; Câmara and Danielsen, 2020; Duman et al., 2021). In contrast to this multifaceted understanding, recent studies in music perception and cognition agree on a sharper definition of groove as the pleasurable urge to move one’s body in relation to the rhythm of music (Madison, 2006; Janata et al., 2012; Stupacher et al., 2013; Senn et al., 2020). These different approaches to defining groove are discussed, for example, by Câmara and Danielsen (2020), who distinguish three aspects of groove: (1) a rhythmic pattern and performance, (2) a pleasurable urge to move, and (3) a state of being.

Here, we focus on the pleasurable, movement-inducing aspect of groove. In this approach, groove is genre-independent, in that every rhythmic pattern and performance evoking the pleasurable urge to move possesses the quality of groove. Although soul, disco, funk and related genres may be more likely to induce groove, the pleasurable urge to move can also be experienced while listening to rock, jazz, electronic dance music, and many other genres.

We argue that groove, when defined as the pleasurable urge to move to a rhythm, depends on a fine-tuned interplay between predictability and surprise. The predictability arises from repetitive rhythmic patterns, and the surprise arises from slight deviations from these patterns, for example in the form of syncopation. This tension represents the sweet spot of the groove experience, and is commonly found in rhythmic patterns that are simple enough for us to interpret and predict, but complex enough to keep us challenged and engaged (Vuust and Witek, 2014; Witek et al., 2014). We will use this perspective to discuss the experience of groove in body, brain, and social interactions.

## Groove in body and brain

The neural mechanisms that are engaged from the moment we start to listen to music to the moment we are tapping our foot in time with the beat or start dancing, rely on the human brain’s ability to integrate external stimuli with internal representations, expectations, or predictions (Koelsch et al., 2019; Pando-Naude et al., 2021). This continuous flow of stimulus-driven bottom-up information and top-down processes requires specialized neural processing, such as audio-motor coupling (Jäncke, 2012), a phenomenon driven by temporal predictions (Vuust et al., 2009; Schröger et al., 2015) that is associated with reward, pleasure, and other cognitive and emotional mechanisms (Koelsch, 2010, 2014, 2020; Koelsch et al., 2013; Salimpoor et al., 2015). How accurately we can predict a rhythm and how pleasurable a rhythm is, therefore depend, on the one hand, on an individual’s long-term priors, such as listening biography, cultural background, musical expertise, dance training, and general cognitive and motor abilities, and on the other hand, on the rhythm’s complexity. Both aspects are integrated in the predictive coding framework (PC), which proposes that the brain minimizes prediction errors by using Bayesian inference when comparing a real-time internal model to a given sensory input (Friston, 2005). When listening to music, this means that we constantly check and update the predictive model of a rhythm by comparing it to the actual musical input (Vuust and Witek, 2014).

### Groove and predictive processing

Within the context of groove, PC has predominately been deployed to interpret the inverted U-shaped relationship between groove ratings and the degree of rhythmic complexity (Vuust and Witek, 2014; Vuust et al., 2018; Koelsch et al., 2019). Rhythmic complexity can depend on different factors, such as meter or microtiming, but is most often operationalized as syncopation, which is when notes occur on weak metrical positions, and are followed by silences on stronger metrical positions (Longuet-Higgins and Lee, 1984). The inverted U shape suggests that moderately syncopated rhythms elicit the strongest sensation of groove (Sioros et al., 2014; Witek et al., 2014; Matthews et al., 2019; Stupacher et al., 2022). Under PC, this effect arises from the fact that moderately syncopated rhythms give rise to the greatest number of strongly weighted prediction errors. Prediction errors result from a mismatch between an internally generated model—here, the beat and meter—and the sensory input (Friston, 2005). Together, beat and meter form a predictive scaffold that determines how strongly we expect a note to occur at each time point (Vuust and Witek, 2014).

**FIGURE 1:**
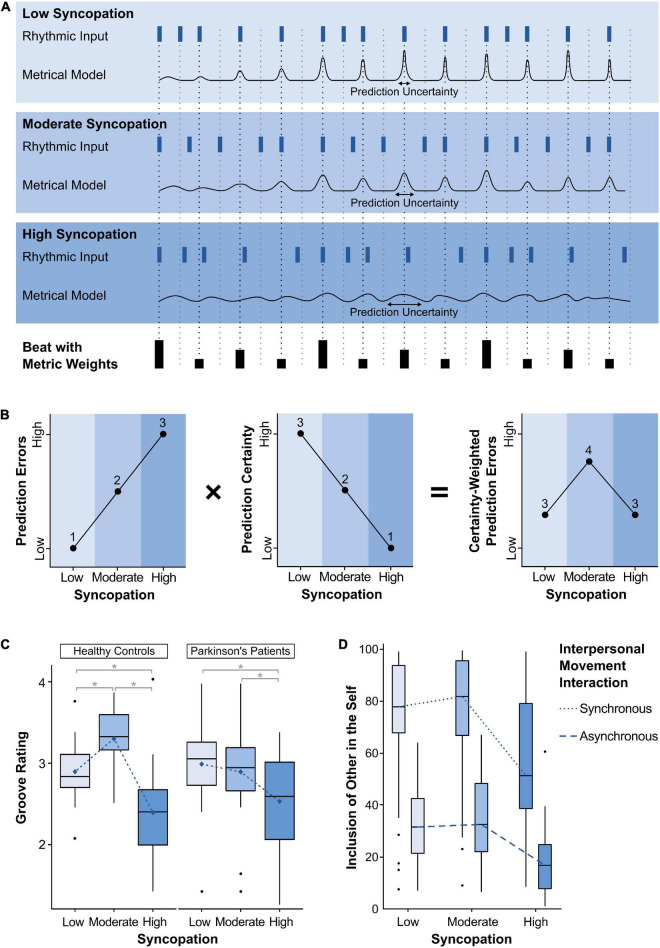
**(A)** Beat-based predictions, prediction uncertainty, and prediction errors for three rhythms with low, medium, and high degrees of complexity, taken from Matthews et al. (2019). Dark blue rectangles denote the onsets of the rhythms and black rectangles denote the underlying beat with the height indicating the metric weight of each beat point according to (Longuet-Higgins and Lee, 1984). The black traces represent metrical models with beat-based predictions delineated as probability distributions wherein the mean of the distribution reflects the predicted onset times, and the width of the distribution reflects the certainty of that prediction. As can be seen across the three traces, the predictions and their certainty depend on the degree of syncopation, the metric weights of each beat, and the progression through the rhythm. That is, prediction accuracy and certainty start out relatively low for all three rhythms as it takes several onsets before a beat and meter is induced. Meter-based predictions can occur for each metric level relevant to a given rhythm, and depending on musical training (Palmer and Krumhansl, 1990), however, for simplicity, only beat-based predictions at the quarter note level are shown. **(B)** The inverted U shape arises from the product of the number of prediction errors and prediction certainty. Prediction errors increase from low syncopation rhythms to high, while the degree of prediction certainty decreases. Multiplying these functions reveals that moderately syncopated rhythms elicit the greatest number of strongly weighted prediction errors. **(C)** Groove ratings in Parkinson’s disease patients (*N* = 24) and healthy individuals (*N* = 27) from Pando-Naude et al. (in preparation). The inverted U-shaped relationship is shifted from moderately complex rhythms in healthy individuals, toward less complex rhythms in PD patients. Blue diamonds indicate mean values. Asterisks indicate significant differences (*p* < 0.05) in pairwise comparisons adjusted with the Tukey method. Boxplots: The centerline represents the median. The lower and upper ends of the boxes correspond to the first and third quartiles. Whiskers represent lowest and highest values within 1.5 × interquartile range (IQR) from the lower and upper quartiles, respectively. Dots represent values outside 1.5 × IQR. **(D)** The tendency of an inverted U shape in relation to the level of syncopation can also be found in social bonding with another person, as measured by Inclusion of Other in the Self (Stupacher et al., 2020).

Panels A and B of Figure 1 illustrate how beat- and meter-based temporal predictions can be conceptualized as probability distributions (Large and Jones, 1999; Danielsen et al., 2019; Koelsch et al., 2019; Cannon, 2021), with their mean and spread reflecting the accuracy and certainty of these predictions, respectively. Prediction certainty determines the weight of the prediction error, that is, the degree to which it affects the metrical model. Syncopations violate meter-based predictions, and thus can introduce uncertainty into the metrical model and the subsequent predictions. Therefore, all else being equal, the degree of syncopation in a rhythm determines both the number and certainty of prediction errors. As shown in panels A and B of Figure 1, moderately syncopated rhythms combine a moderate number of prediction errors with a moderate degree of certainty. They therefore elicit the strongest top-down engagement to update and maintain the metrical model, that is, to minimize further prediction errors and uncertainty.

According to PC, there are two ways to minimize prediction errors and thereby reduce uncertainty (Friston, 2003). One way is to change the model to better fit the input, either by shifting the phase of the meter, i.e., ‘resetting,’ to better align with the rhythm (Fitch and Rosenfeld, 2007), or switching to a different meter altogether, e.g., from a duple to a triple meter. Recent empirical and theoretical work implicates the motor system in the phase resetting and dynamic maintenance of temporal predictive processes during rhythmic auditory tasks (Morillon and Baillet, 2017; Rimmele et al., 2018). The second way to minimize prediction error is to move one’s body in time with the beat (Koelsch et al., 2019). Moving to the beat results in rhythmically timed proprioceptive input that reinforces the metrical model. Therefore, by optimally challenging the predictive model, moderately syncopated rhythms lead to greater engagement of the underlying motor systems, which is experienced as an automatic urge to move.

Uncertainty reduction also engenders the pleasurable component of groove. Within PC, this uncertainty reduction, as driven by improving the correspondence between model and input, corresponds to learning. In this context, learning is thought to be inherently rewarding as it serves the innate drive for competence (Ryan and Deci, 2000) and satisfies the intrinsic motivation for information gain (i.e., curiosity; Schmidhuber, 2010; Kidd and Hayden, 2015; Friston et al., 2017; Gottlieb and Oudeyer, 2018). Therefore, organisms are intrinsically motivated to seek out and attend to activities that afford maximal uncertainty reduction, in other words “learnable activities that are just beyond [their] current predictive capacities” (Oudeyer et al., 2016, p.9). In this context, moderately syncopated rhythms elicit pleasure by maximizing the intrinsic reward derived from actively refining our predictive processes *via* covert or overt motor processes. Recent work has demonstrated a direct link between music-evoked reward and moderate levels of prediction error and uncertainty in the context of melodic and harmonic expectations (Cheung et al., 2019; Gold et al., 2019b; Shany et al., 2019). We believe that applying a similar approach to groove will be crucial to understanding why a rhythmic sweet spot has such power to move us, and to uncovering the neural mechanisms that drive this effect.

Intriguingly, the basal ganglia (BG), a set of subcortical nuclei involved in motivation and motor control, along with dopaminergic transmission within the BG, have been implicated in all of the processes discussed above, including beat and meter-based perceptual and motor timing (Schubotz et al., 2000; Grahn and Brett, 2007; Grahn and Rowe, 2009; Schwartze et al., 2011; Kung et al., 2013), music-evoked pleasure (Salimpoor et al., 2011, 2013; Gold et al., 2019a), and prediction certainty (Friston et al., 2014; Owens et al., 2018; Gershman and Uchida, 2019). This suggests that the BG and dopamine play a crucial role in groove, a perspective that is supported by a recent fMRI study linking groove ratings to activity within limbic- and motor-associated BG nuclei (Matthews et al., 2020). Based on these results, the authors proposed a theoretical model wherein beat-based temporal predictions and the associated reward are integrated in the BG *via* parallel striato-cortical loops, particularly the limbic, motor, and associative loops (Alexander et al., 1986; Obeso et al., 2008).

### Groove and Parkinson’s disease

One way to test the role of the BG and dopamine in groove is to compare Parkinson’s disease (PD) patients with healthy controls. PD results from dopaminergic dysfunctions in the BG caused by neuronal degeneration of the substantia nigra pars compacta (SNc). Such dopaminergic depletion disrupts disinhibitory mechanisms between the BG and the motor thalamus, altering the fine-tuning between initiation and suppression of the activity in the motor loop, and giving rise to the characteristic motor symptomatology. In terms of groove, a recent study showed that the inverted U-shaped relationship between rhythmic complexity and pleasurable desire to move is shifted from moderately complex rhythms in healthy individuals, toward less complex rhythms in PD patients, who seem to prefer little incongruence between the internal predictive model and the stimulus (Figure 1C; Pando-Naude et al., in preparation). Notably, PD patients do not show an overall reduction in groove ratings, suggesting that PD does not reduce the overall urge to move to the rhythm, but only alters which types of stimuli elicit these responses, potentially as a function of altered predictive processes.

Research into auditory-motor activity and the neural correlates of rhythm perception (Schubotz et al., 2000; Grahn and Brett, 2007; Chen et al., 2008; Bengtsson et al., 2009; Grahn and Rowe, 2009; Grahn, 2012) has led to new approaches for developing movement therapies. Rhythmic auditory stimulation improves motor deficits in patients with PD by providing a regularly-timed cue, such as a metronome, with which patients can synchronize their gait (Thaut et al., 1996; Pau et al., 2016; Dalla Bella et al., 2017; Lei et al., 2019). However, the low rhythmic complexity of the metronome may restrict the method’s benefits (Dalla Bella et al., 2015; Cochen De Cock et al., 2018). In contrast, ecologically valid stimuli incorporating both rhythmic and harmonic elements may lead to a richer set of predictions, potentially promoting better guidance for temporal models of movements (Vuust et al., 2014). Dancing might offer an even more ecologically valid and rich PD intervention that can improve gait symmetry, decrease dual task costs (Fontanesi and DeSouza, 2021), and reduce disease severity (Krotinger and Loui, 2021). As discussed above, moderately complex stimuli are likely to increase engagement of motor timing and reward processes involving the BG (Matthews et al., 2020), potentially boosting motor benefits by increasing dopaminergic signaling in these nuclei (Salimpoor et al., 2013; Hansen et al., 2017; Matthews et al., 2020). A challenge for future research on music-supported movement and dance therapies is to investigate whether an individual PD patient’s preference for a certain level of rhythmic complexity may depend on the progression of the disease. One objective of future studies could therefore be to find individualized sweet spots of rhythmic complexity for PD patients with different levels of auditory, sensorimotor, and timing deficits.

By comparing the experience of groove in PD patients and healthy controls, we contribute to our understanding of the underlying neural mechanisms linking temporal predictions, movement, and reward. In turn, a better understanding of these mechanisms can guide future approaches to better treat motor deficits, and improve the quality of PD patients’ personal and social life.

## Groove in social interactions

When people come together to listen to or make music, the level of rhythmic complexity that hits an individual’s sweet spot for an optimal groove experience depends on their biology and cultural background. In his interviews with musicians, Charles Keil noted that “each person has a unique feel for time and that bringing different or discrepant personalities together generates different kinds of groove” (Keil, 1995, p.8). Keil calls the intentional deviations in timing that result from the constant relating and negotiating between players *participatory discrepancies* and hypothesizes that they are necessary to make music involving and socially meaningful (Keil, 1987, 1995). This type of involvement may be especially strong with syncopated rhythms, as performers—live or recorded—can invite listeners and dancers to participate in the relating and negotiating by filling in the gaps in the syncopated rhythmic structure (Witek, 2017).

### Groove and dance

Music and dance are so intertwined that some cultures do not distinguish between them (Haugen, 2021), and groove is an important element in understanding this connection (Foster Vander Elst et al., 2021). Fitch argues that “if we want to understand the rhythmic origins of a musical style, it behooves us to know how contemporaries would have moved to that music” (2016, p.6). Dance is also an intrinsically social activity that encourages social bonding (Tarr et al., 2014, 2015; Launay et al., 2016). Indeed, compared to synchronizing movements in silence or with a metronome, music can increase social closeness with another person (Stupacher et al., 2017, 2021). Furthermore, the number of people who can easily converse together is usually limited to four (Robertson et al., 2017), but much larger groups regularly form when people dance together. It has therefore been posited that the prosocial and emotional effects of group music-making and dancing might be evolutionary adaptations (Huron, 2001; Loersch and Arbuckle, 2013; Trainor, 2015).

From the perspective of predictive processing, beat and meter are mental models that can be shared by all dancers and musicians in a group. If the models are alike, i.e., ‘distributed’ across dancers, they facilitate synchronous movements, which in turn can promote shared affective experiences (Witek, 2019). In addition to strict synchronization, dance is commonly also concerned with the expression of creativity and “individual flourish” (Merker et al., 2009) within the framework of a mental model of beat and meter. When dancing together, moderately syncopated rhythms may provide the ideal stimulus for facilitating both united synchrony and individual creativity. On the one hand, moderately syncopated rhythms include enough notes on strong metrical positions that allow the dancers to form shared beat- and meter-based predictions. On the other hand, these rhythms also include notes on weak metrical positions, “injecting energy” into upbeats (Fitch, 2016), and pauses on strong metrical positions, inviting dancers to fill the “open spaces” with creative movements (Witek, 2017). Therefore, moderately syncopated rhythms may provide a common temporal framework within which dancers can share basic movements, but also inspire each other with individual creative movements—a combination that may facilitate the experience of shared emotions. This perspective is supported by a recent study suggesting that social bonding with another person tends to follow an inverted U shape in relation to the degree of syncopation (Figure 1D; Stupacher et al., 2020). A certain level of complexity is also preferred when playing the Mirror Game, in which two individuals move as coordinated and synchronously as possible (Ravreby et al., 2022). Ravreby and colleagues found that social bonding with the partner increases with increasing interpersonal synchronization, but also with movement complexity. Although simple movements benefit from greater synchronization accuracy, more complex and novel movements may be introduced to keep each other interested and engaged.

Both dancing and performing music in a group involve moving in time together. However, dance and music improve sensorimotor integration in both shared and unique ways (Giacosa et al., 2016, 2019; Karpati et al., 2017). For example, musicians perform better than dancers when synchronizing finger taps with auditory rhythms, whereas expert dancers perform better than musicians when imitating whole-body dance movements (Karpati et al., 2016). To date, most of the research investigating long-term-priors, such as the effects of expertise and listening biographies on groove is concerned with effects of musical training and preferred musical style. Musicians show greater neural activity in motor-related areas of the brain when listening to high-groove music (Stupacher et al., 2013), and a more pronounced U-shaped relationship between groove ratings and degree of syncopation (Matthews et al., 2019, 2022). Additionally, Senn and colleagues note that “listeners’ susceptibility to bodily entrainment as a response to music is strengthened when the music agrees with their taste” (Senn et al., 2018, p.26). The rare studies on groove that have tested the effect of dance experience are limited to behavioral paradigms, which suggest that participants with greater frequency and enjoyment of dancing experience more groove (Witek et al., 2014; Matthews et al., 2019). However, while both of these studies differentiate sharply between musicians and non-musicians based on years of training, the differentiation between dancers and non-dancers is less clear. Given the unique ways in which dancing affects whole-body sensorimotor integration (Giacosa et al., 2016, 2019), it would be beneficial for future work on the experience and neurophysiology of groove to use similarly sharp selection criteria for dancers. Groove is a universal phenomenon, but some effects may be more pronounced in experienced dancers, who are experienced in using their entire bodies to actively and creatively engage with music.

## Conclusion

In dance, music making, and music listening, groove research can help us to better understand the interactions between temporal processing, movement, social behavior, and pleasure in both the general population and individuals with motor impairments. Elucidating the neural mechanisms underlying groove—especially the role of the basal ganglia—will contribute to our understanding of the integration of motor driven predictive timing and reward processes more generally.

One open question is how the experience of groove differs when comparing individual versus collective situations of music listening or dancing. It is also unclear how the type of rhythm-related movements affects groove experiences. Future research could, for example, compare whole body engagement in dance with specific movements of particular body parts, such as finger tapping or playing an instrument. Another future direction of groove research could be to investigate activities outside the field of music. Senn and colleagues define the verb to groove as “playing music together in an effortless and rhythmically well-coordinated manner” (Senn et al., 2020, p.46). This use of groove can also be applied to other coordinated interindividual activities, such as playing team sports, verbal communication, or gestural mimicking. Based on the relationship between rhythmic complexity and groove in music, it could be expected that in these activities rhythmic complexity follow similar U-shaped functions when measuring pleasure and engagement.

## Data availability statement

The original contributions presented in this study are included in the article, further inquiries can be directed to the corresponding author.

## Author contributions

JS: conceptualization and writing – original draft, review and editing. TEM, VP-N, and OVFE: writing – original draft, review and editing. PV: writing – review and editing. All authors contributed to the article and approved the submitted version.
